# Perception towards reducing natural gas consumption and imports in Europe: A theoretical and empirical investigation

**DOI:** 10.1016/j.heliyon.2024.e30719

**Published:** 2024-05-03

**Authors:** Javanshir Fouladvand, Francesco Fiori, Özge Okur

**Affiliations:** a Copernicus Institute of Sustainable Development, Utrecht University, the Netherlands; b Human Geography and Spatial Planning Department, Faculty of Geosciences, Utrecht University, the Netherlands; c Multi-Actor Systems Department Technology, Policy and Management Faculty, Delft University of Technology (TU Delft), the Netherlands

**Keywords:** Energy behaviour, Natural gas, Theory of planned behaviour, Energy transition, Energy supply, Survey

## Abstract

The European energy system is undergoing a drastic change, focusing on reducing natural gas consumption and import. European households, which are responsible for 25 % of final energy consumption, mainly based on natural gas, could play a significant role in such a transition. Therefore, the study aims to understand the perception towards natural gas consumption reduction and imports. An online questionnaire was designed based on an extended version of the theory of planned behaviour, which 257 highly educated respondents filled out. The results delineated the respondents' positive perception of reducing natural gas consumption. Specific attributes (i.e., environmental concerns, national sustainable and efficient energy system, and national energy independence) and personal moral norms (i.e., moral responsibility) significantly impact the willingness and effort to reduce natural gas consumption. The lack of control is the largest perceived control behaviour in reducing the respondents' natural gas consumption compared to available knowledge and affordability. Finally, the respondents care significantly and want to know about natural gas import sources, and they are highly against natural gas imports from Russia, the United States, the Middle Eastern, and Arab countries. Based on the insights, the study provides detailed recommendations. The study provides concrete recommendations for policy-makers to include environmental, humanitarian and energy-independence concerns in their decision-making processes related to natural gas imports and consumption. It also emphasises informing and involving individual households in such decision-making processes.

## Highlights

• Perception towards natural gas consumption and imports was investigated.
• A survey was built based on an extended version of the Theory of Planned Behaviour.
• Respondents were considerably positive towards reducing natural gas consumption.
• Lack of control is seen as the main barrier to reducing natural gas consumption.
• Humanitarian concerns are crucial in choosing the source of natural gas imports.

## 1 Introduction

Fossil fuel consumption reduction is one of the key challenges for the energy transition, as different sectors, such as industries, transport and households, heavily rely on fossil fuel consumption [1]. Different countries and regions developed various targets to reduce fossil fuel consumption and ultimately contribute to reducing greenhouse gas (GHG) emissions [2]. Particularly, the European Union (EU), as one of the main actors in the energy sector and energy transition, has developed specific targets and strategies [3].

Energy strategies have always been one of the main topics in EU policy [4], as it is a net energy importer [5,6]. In this context, EU households are responsible for approximately 25 % of total energy consumption in the EU [3,7]. In particular, approximately 70 % of this consumption is due to natural gas consumption for heating purposes [7]. Therefore, considerable natural gas consumption resulted in high GHG emissions from EU households [8]. Their consumption also reflects heavily on natural gas imports, as according to different data sets, approximately 60 % of the European gas demand was covered by imports in 2018 [9], mainly from Russia [10]. However, due to the ongoing crisis in Eastern Europe and its consequences on the energy market [10], the EU has announced new targets for reducing natural gas consumption and phasing out Russian natural gas imports [11]. Thus, along with the need to reduce the environmental impact of EU households (e.g. GHG emissions), such geopolitical crises and energy security issues highlighted the urgency of reducing EU households' natural gas consumption.

Various studies have focused on alternative energy systems as solutions for addressing the shift from natural gas consumption in the EU residential sector. For instance, studies such as [12,13] explored different EU geothermal energy scenarios. Different studies focus on geothermal energy in a specific country (e.g. Ref. [14] in the Netherlands and [15] in Finland). The potential and scenarios for district heating in the EU are demonstrated in Ref. [16]. The techno-economic feasibility of Denmark's district heating and heat pumps as an alternative solution for heating residential areas are presented in Refs. [17,18]. An overview of district heating in the Swedish context is provided in Refs. [19,20]. Policy interventions and business models related to English district heating are studied in Ref. [21]. On the other hand, the thermal energy transition in the residential sector of China, Denmark, Finland and the United Kingdom is presented and compared in Ref. [22].

In addition to these studies, various studies explored the EU households' attributes and behavioural changes in the context of energy transition. For instance, the influence of households' environmentally friendly behaviour on greenhouse gas emissions and climate change mitigation in the European residential sector is investigated in Ref. [23]. By applying different behavioural theories, the factors influencing space heating and energy consumption in the EU are explored in Ref. [24]. The influence of environmentally friendly behaviour on the energy security of such collective energy systems is demonstrated in Ref. [25]. The influence of behavioural patterns and user profiles on heating consumption is explored in Ref. [26]. Investigating the behavioural and socioeconomic factors and their impact on Dutch households' energy consumption, including heating purposes, is presented in Ref. [27]. Various studies, such as [28,29], discussed the factors influencing the individual's participation in the local (heating) energy transition. Along with technical and institutional conditions, participants' behaviour in successfully establishing local collective heating systems is explored in Refs. [30,31]. However, such studies focus on alternative solutions (e.g., energy efficiency measures and renewable energy systems) and do not specifically explore the willingness to make behavioural changes in reducing natural gas consumption. More importantly, none of these studies has addressed such topics in the context of the EU influenced by the ongoing energy crisis. Furthermore, no study explores opinions and perceptions on European natural gas import sources. Considering the ongoing climate and geopolitical crisis, studying and understanding such topics are vital for Europe and its energy transition.

To address this gap, this study explores European households' perception of reducing natural gas consumption and imports. Therefore, the study sets the following research question: What is the perception of individual households in Europe on natural gas consumption and imports? The study empirically approaches its aim (and research question) by collecting and analysing data through an online questionnaire. The theory of planned behaviour (TPB) [31] is employed as a theoretical basis to constructively and systematically explore the EU households' intentions and behaviour. The TPB is developed explicitly for studying particular behaviours, and several studies, such as [32,33], have already been applied to analysing energy-related behaviours. The data collection is focused on highly educated households to address the research aim constructively. Individuals with a high willingness to change their behaviour (e.g., early adopters of energy innovations and, in this case, reducing natural gas consumption) are among the higher-educated individuals [34,35]. This can be translated as such a sample could be more representative of individuals who potentially are willing to change their behaviour. By such a systematic approach, the study contributes to the literature on energy-related behaviour and energy policy.

The study also aims to provide concrete insights and recommendations to relevant actors in decision-making processes. The insights could potentially be seen as the (highly educated) households' opinions and, therefore, increase their voice in decision-making. In addition, such insights and recommendations could also potentially contribute to the energy policy at a higher level (such as national, European and International Energy Agency (IEA) levels) to reduce natural gas consumption, make changes in natural gas import, and achieve energy transition goals. More specifically, this study can be seen as a response to concerns in relation to the energy crisis caused by the ongoing war in Eastern Europe, as demonstrated in studies such as [36,37]. To summarise, the contributions of this work can be seen in three main points.

❖ To explore the perception of highly educated individuals towards reducing natural gas consumption for the first time after the crisis in Eastern Europe;
❖ To understand the preferences of highly educated individuals towards alternative natural gas exporters for the first time to include individuals' perspectives on high-level energy policy;
❖ To apply the theory of planned behaviour on individuals' perception towards a national-level energy-related issue (i.e., natural gas consumption and imports) for the first time.

The structure of the paper is as follows. Section 2provides the theoretical framework. Section 3 elaborates on research methods. Section 4 presents the results. Discussions and recommendations are presented in Section 5. Section 6 demonstrates the conclusions and final remarks.

## 2 Research approach

This section first presents the theory of planned behaviour as the theoretical background. Then it demonstrates the online questionnaire as the research method to collect data and investigate the perception towards reducing natural gas consumption and imports in Europe.

### 2.1 Theoretical framework: theory of planned behaviour

**Fig. 1:**
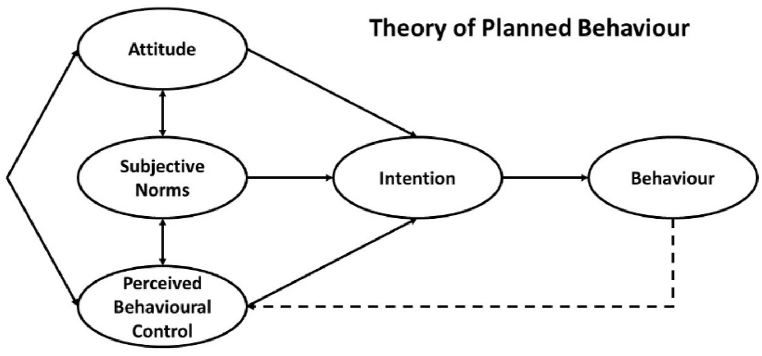
Theory of planned behaviour (TPB) [38].

The theory of planned behaviour (TPB) is a conceptual framework dealing with the complexities of human social behaviours [38]. The TPB aims to understand and predict particular behaviours in specified contexts [38]. The framework focuses on behavioural intentions, as they can account for a considerable proportion of variance in behaviour [39]. To understand and predict such intentions and behaviours, the framework groups the determinants of intention for certain behaviours into three main conceptually independent components, namely (i) attributes toward the behaviour, (ii) subjective norms with respect to the behaviour, and (iii) perceived control behaviour over the behaviour, as presented in Fig. 1.

Further elaboration on each component of the TPB is as follows.

❖ Attitudes: refers to the degree to which a person has a (un)favourable evaluation of the behaviour, which is related to the behavioural beliefs;
❖ Subjective norms: refers to the social pressure to (not to) perform the behaviour, which is related to social factors;
❖ Perceived control behaviour: refers to the perceived level of difficulty in performing the behaviour, which is assumed to reflect on past experience as well as anticipated impediments and obstacles.

In the realm of energy transition, various studies employed the TPB to study specific energy-related behaviours. For instance, the prediction of willingness to pay for energy transition has been studied in Ref. [40]. To study the intentions to mitigate climate change, an exploration of the energy savings and carbon reduction behaviour of individuals in Taiwan by employing the TPB is presented in Ref. [32]. Studies such as [33] explore the intentions of Iranian households to invest in renewable energy projects. In another similar study, presented in Ref. [41], the influential factors for intention to invest in a community-owned renewable energy initiative in Australia are studied. The intention to participate in community energy systems is explored in Ref. [42]. By using an extended version of the TPB, the individuals' energy-saving behaviours in the working environment are studied [43]. The relationship between organisational interventions and employees' energy-saving behaviours is studied in Ref. [44]. By applying the TPB [45], demonstrates the influential factors for accelerating the adoption of shared electric cars. To predict the consumers' intention to adopt hybrid electric vehicles [46], also applied the TPB to the data collected from individuals in China. However, none of such studies have focused on reducing natural gas consumption in the EU context.

In the current study, following studies such as [32,43,46], an extended version of the TPB is used. In the extended version, along with its initial three components, two other ones are added: personal moral and descriptive norms. Personal moral norm refers to individuals' behaviours based on their moral responsibility or obligation [47]. As it has been known that the behaviour of someone has a strong influence on an individual's behaviour [48], descriptive norms reflect on what a significant number of individuals actually do and then a particular individual thinks he/she should do [49]. As argued in the mentioned studies, extending the TPB with these two components would provide a broader understanding of the intentions and behavioural aspects. Therefore, the following five components will be used to understand the intentions for reducing natural gas consumption: (i) attributes, (ii) subjective norms, (iii) perceived control behaviour, (iv) personal moral norms, and (v) descriptive norms.

### 2.2 Research method: online questionnaire

The research method is the statistical analysis based on empirical data collected from an online questionnaire conducted among EU countries, specifically focusing on the Netherlands. This section briefly introduces the questionnaire and questions used in this research. Further detailed information can be found in Appendix A. The questionnaire is in line with the European General Data Protection Regulation (GDPR) guidelines (e.g., the respondent is well informed about the purpose of the questionnaire and the data gathering and storage process, and they should be older than eighteen years old).

The questionnaire consists of three parts: (i) the socio-demographical data, (ii) current energy systems and consumption, and (iii) intentions for reducing natural gas consumption. The first part includes socio-demographic indicators such as income, education, housing situation and the number of people in a household. The second part includes information on energy bills, the current type of energy system, and its satisfaction. For the last part, following studies such as [32,43,46], 20 questions aimed at understanding behaviours and perceptions towards natural gas are developed, which consist of statements that the respondent must rate based on the level of agreement/disagreement for each statement. These statements are on a 5-Likert-type scale and linked to five components of the TPB, as presented in Section 2. Therefore, by analysing the results of these statements, the impact of components on the intentions for reducing natural gas consumption is explored. These statements and the results are presented in Appendix A and Section 4.2. In the end, there are also questions regarding the independence and source of national gas imports.

The results are presented in two main batches: (i) results from all the European respondents (presented in Sections 4.1. and 4.2.), and (ii) a comparison of the results from Dutch respondents from the rest of the European respondents (presented in 4.3.). As explained in Section 1, the study is structured to approach the intentions for reducing natural gas consumption by focusing on European countries. As the Netherlands has unique characteristics within the European context, comparing the results from the Dutch perspective and the rest of the European respondents could potentially bring more insights. Some of the Dutch energy systems' unique characteristics are as follows.

❖ Available large natural gas field and is a natural gas hub in Europe [50,51];
❖ Dutch national ambitious CO_2_ reduction targets which influenced the heating sector [52];
❖ Gas quakes and their influence on natural gas consumption and energy transition [53];
❖ Actors' conflicting opinion towards natural gas [54,55];
❖ Strong energy security performance of the Netherlands and its importance in the Dutch energy policy [56,57].

Further elaboration on the unique energy system of the Netherlands, particularly on the natural gas background in the Netherlands, is presented in Refs. [58,59], and [25].

## 3 Results

The questionnaire was performed between the beginning of January 2023 and the beginning of March 2023 (for 54 days in total) using an online questionnaire collector tool of Qualtrics. 350 individuals from EU countries approached the online questionnaire, of which 257 were completed. The response rate is 73 % overall. The socio-demographic characteristics of the respondents are summarised in Table 1.

**Table 1:** Socio-demographic information.

QUESTION	Answers	Number	Percentage
Total respondents		257	100
Gender	Male	141	54.9
	Female	110	42.8
	Non-Binary	3	1.2
	Prefer not to say	3	1.2
	Others	0	0.0
Age	18–24	27	10.5
	25–34	126	49.0
	35–49	68	26.5
	50–64	28	10.9
	≥ 65	8	3.1
Level of education	No degree	0	0.0
	High school	2	0.8
	Professional training	2	0.8
	Undergraduate	40	15.5
	Master	132	51.4
	PhD	81	31.5
Employment status	Part-time	19	7.4
	Full-time	181	70.4
	Unemployed	6	2.3
	Retired	3	1.3
	Student	43	16.7
	Others	5	1.9
Household status	Only me	63	24.5
	Family house (2–5 people)	141	54.9
	Shared house (2–5 people)	44	17.1
	Family house (>5 people)	0	0.0
	Shared house (>5 people)	9	3.5
Localisation	Urban city centre	121	47.1
	Urban periphery	118	45.9
	Rural area	18	7.0
Level of income compared to the average of the country	Much lower	57	22.2
	Lower	55	21.4
	Average	43	16.7
	Higher	74	28.8
	Much Higher	28	10.9

As Table 1 presents, the respondents were almost evenly distributed among men and women. Most respondents were between 25 and 34 years (49 %); 26.5 % were between 35 and 49 years, 10.9 % were between 50 and 64 years, 10.5 % were between 18 and 24 years, and 3.1 % were above 65 years. 51 % of the respondents were European people living in the EU, while 49 % were not originally European. Most respondents have a university degree (31.5 % PhD + 51.4 % MSc + 15.5 BSc = 98.6 %), which aligns with focusing on highly educated households as the focus of this study.

The majority of the respondents were working full-time (70,4 %), 16.7 % were students, and 7.4 % were working part-time. The respondents mainly lived with their families (54.9 %), then lived alone (24.5 %), and then lived in a shared house (20.6 %). 93 % of the respondents were living in an urban area (either city centre or periphery/suburb), while only 7 % were living in rural areas. As far as household-level income is concerned, the respondents are scattered in different income categories. 16.7 % reported income in the average of the country of their residence, 28.8 % higher and 10.9 significantly higher than the country of their residence. On the other hand, 21.4 % reported having lower incomes, and 22.2 % reported having much lower incomes compared to the average income in the country of their residence.

Although the sample is limited to 257 respondents and it is biased towards highly educated individual households with a full-time job, analysing the collected data could be insightful, particularly from a theoretical perspective, to understand the overall influence of the TPB components on the desired behaviour, in this case, reduction of natural gas consumption within highly educated individuals.

### 3.1 Perception towards the current energy system

As presented in Table 2, the respondents were asked to indicate variables related to the techno-economic information on their current energy systems.

**Table 2:** Techno-economic information on the current energy systems.

QUESTION	Answers	Number	Percentage
Total respondents		257	100
**Energy bills**	<5 %	91	35.4
	5–10 %	68	26.5
	10 o 15 %	38	14.8
	15–20 %	9	3.5
	>20 %	6	2.3
	Idk	45	17.5
**Type of current heating systems (multiple answers were possible)**	National grid, gas	189	59.8
	Individual renewable	31	9.8
	District renewable	24	7.6
	others	23	7.3
	I do not know	23	7.3

The majority of respondents (approximately 61.9 %) spend less than 10 % of their monthly income on their energy bills. However, 20.6 % of respondents spend more than 10 % of their monthly income on their energy bills, which, based on the ten percent rule index (TPRI) such percentage is considered an energy-poor household [58,59]. This includes 2.3 % of respondents (i.e., 6 respondents) who spend more than 20 % of their income on energy bills. Considering the highly educated sample (which could potentially be entitled to higher and sustained income), such a high percentage of energy poverty is concerning and unsustainable in the long run.

From the energy resource and technological point of view, most respondents use natural gas for their heating purposes (59.8 %), which shows that most of them rely on natural gas consumption. 17.4 % of respondents already use some type of renewable energy technologies, either collective or individual technologies (e.g. district heating, heat pump and solar thermal). Among the respondents, 7.3 % did not know what type of heating systems they use in their household, which can be interpreted as their lack of importance and interest in such topics, who are speculated to be mainly tenants. Considering this information, the respondents were asked to indicate their perception towards the current energy systems, as presented in Table 3.

**Table 3:** Perceptions towards the current energy systems.

Statements	The average score of 257 EU respondents on the 5-Likert-type scale
**The current heating system is always available.**	4.3
**The current heating system is always affordable.**	3.52
**Due to the current heating system, my accommodation is always comfortable.**	2.78
**The current heating system is environmentally friendly.**	3.46
**I am satisfied with the current heating system.**	2.23

Although the households find their current energy systems mostly available (i.e., 4.3 out of 5 on average) and relatively affordable (i.e., 3.52 out of 5 on average), their satisfaction is significantly low (i.e., 2.23 out of 5 on average). Such low satisfaction could potentially be due to issues related to accessibility (e.g., old infrastructure) and lack of alternative options. Perceptions towards comfort are the second lowest score (i.e., 2.78 out of 5), and eco-friendliness scored 3.46. Such results highlight that availability and affordability are not the main criteria for determining household satisfaction with their heating systems. Considering the perceptions towards environmental friendliness and comfort level of the energy systems sheds light on understanding the low satisfactions; however, other aspects (such as energy independence and energy sources) need to be considered, as elaborated in the next section.

### 3.2 Analysing the components of the theory of planned behaviour

This section presents the data and analysis for each TPB component. As discussed in Section 2, an extended version of TPB is used, which includes five main components to study the intentions and behaviours for reducing natural gas consumption: (i) attributes, (ii) subjective norms, (iii) perceived control behaviour, (iv) personal moral norms, and (v) descriptive norms. Table 4 demonstrates the results of the questionnaire on these components.

**Table 4:** Questionnaire's data related to TPB.

TPB's components	Question tag	STATEMENTS (the full statements are presented in Appendix A)	The average score of 257 EU respondents on the 5-Likert-type scale
**Attitude**			
	Q13-1	Protecting environment	4.13
	Q13-2	Saving money	3.95
	Q13-3	Having continuous energy access	3.65
	Q13-4	Improving the comfort temperature	2.62
	Q13-5	Making my country energy-independent	4.04
	Q13-6	Contributing to my country to have a more sustainable and efficient energy system.	4.32
**Subjective norms**			
	Q14-1	Influence of family and friends	2.76
	Q14-2	Influence of my neighbours	2.62
	Q14-3	Influence of my colleagues	3
	Q14-4	Influence of me on my friends and family	2.74
**Perceived behavioural control**			
	Q15-1	Access to the required knowledge	3.63
	Q15-2	Affordable (money)	2.96
	Q15-3	Having enough time	2.69
	Q15-4	Having control	2.44
**Personal moral norms**			
	Q16-1	I have a moral responsibility	3.88
**Descriptive norms**			
	Q16-2	My family members/friends did	3.42
	Q16-3	My neighbours and fellow citizens did	3.42
	Q16-4	My colleagues did	3.54
**Intention and behaviour**			
	Q17-1	Willingness to	4.19
	Q17-2	I will make an effort to	3.96

First, about the attitudes, six statements are exposed related to six main attributes: environmental concerns, financial concerns, continuous access concerns, comfort concerns, national independence concerns, and national sustainability and efficiency concerns. As Table 4 shows, among these six statements, the national sustainability and efficiency concerns is the ones that scored significantly highest. It also scored the highest among all the variables, highlighting the importance of national sustainability and efficiency concerns in individuals' energy-related decisions. After that, environmental and national independence concerns scored the highest (higher than 4). The statement about improving the comfort temperature scored the lowest (i.e., 2.62), showing the respondents' least interest in such an attribute.

In the subjective norms component, four statements are exposed, namely related to including (i) family and friends, (ii) neighbours, (iii) colleagues and (iv) the respondent's influence. In general, all the statements in this component are scored in the range between 2.50 and 3.00 on average, which is much lower than other TPB components and variables. This observation can be translated into two points: (i) subjective norms overall are not as strongly involved in decisions related to the reduction of natural gas consumption (for further elaboration, also see Section 4.3.), and (ii) the different social circles have almost the same influence on such decisions from the respondents perspective within EU.

As for the perceived control behaviours, four statements related to knowledge, finances, time and having control were included. The access to knowledge scored the highest, showing respondents have or know where to find the necessary information to reduce their natural gas consumption. In contrast, controlling the reduction of natural gas consumption scored the lowest in this component and, in general, among all the variables. This demonstrates that despite overcoming other barriers (e.g., investment costs, knowledge and available technology), the respondents perceive a lack of control over such a decision. For instance, the following is a quote from the questioner: "Don't care. My opinion does not matter anyway".

The four statements related to personal moral norms and descriptive norms are among the highest scores in general. Respondents morally feel responsible for reducing their natural gas consumption (i.e. on average 3.89), while they believe their social circles also reduce their natural gas consumption. Such high scores demonstrate the importance of this component on intentions and behaviours for reducing natural gas consumption (see section correlations for further elaborations).

In general, within the five components of the extended version of TPB, attitudes and personal moral norms have higher scores in comparison with the other three components. As shown in Table 4, the statements related to these two components have higher absolute scores, which can be translated as their importance in the context of natural gas consumption reduction. These results confirm the findings of studies such as [25,32], demonstrating the importance of attitudes for energy-related behaviour. On the other hand, the low scores of statements for the perceived control behaviour component can be translated as a barrier to reducing natural gas consumption.

Finally, as presented in Table 4, respondents have a high willingness to reduce their natural gas consumption (i.e., 4.19 on average). Although respondents show less willingness to make an effort (i.e., 3.96), this score is still high and demonstrates the public's considerable potential to reduce their natural gas consumption. Especially considering the scores in the other questions (e.g. lack of control and affordability), the willingness of respondents to reduce their natural gas consumption is significantly high. Such households with higher willingness than their effort could be seen as a starting point to motivate effortless households to reduce their natural gas consumption. This difference could be related to the influence of different variables, such as affordability or lack of control over such decisions. However, further research is needed to draw more concrete results.

### 3.3 Correlations

After presenting the absolute scores in the previous sections, this section discusses the correlation of the different variables with willingness (Question tag: Q17-1) and making an effort (Question tag: Q17-2) to reduce natural gas consumption. Absolute scores could be used to show the importance of a certain statement (and component) for reducing natural gas consumption, while correlations can be seen as to what extent such statement or component impacts such behaviours.

**Fig. 2:**
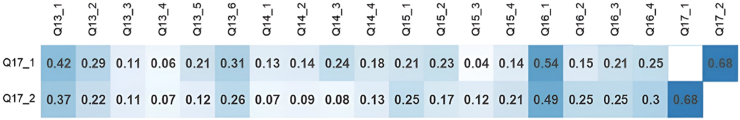
Correlations of different variables with intentions and behaviours (all question tags and statements are presented in Table 4 and Appendix A)

As Fig. 2 presents, the variables with relatively high correlation (equal or higher than 0.30) with both willingness to and making an effort to reduce natural gas consumption, respectively, are one of the personal descriptive norm variables (i.e., moral responsibility, Q16_1) and one of the attributes variables (i.e., environmental concerns, Q13_1). For the willingness to reduce natural gas consumption, national sustainability and efficiency concerns (as an attributes' variable, Q13_6) is the third influential variable, while for making an effort to reduce natural gas consumption, the influence of the colleagues (as a descriptive norm's variable, Q16_4) is the on the third place. Furthermore, the other variables within personal descriptive norms also have relatively higher correlations with making an effort rather than the willingness to reduce natural gas consumption. On the other hand, variables in the subjective norms component have relatively higher correlations with the willingness to rather than make an effort to reduce natural gas consumption.

### 3.4 Comparing the EU and the Netherlands

As discussed in Sections 1 and 3, due to the unique characteristics of the Dutch energy system, the study compares the responses from the Netherlands with the rest of the EU. This comparison contributes to understanding the intentions and behaviours for reducing natural gas consumption while bringing further insights into the Dutch energy transition. Table 5 presents this comparison.

**Table 5:** Comparison between the EU and the Netherlands.

QUESTIONS	STATEMENTS	NL	EU-NL	Difference in percentages^a^
**Number of Respondents**		177	80	
**Attitude**				
	Protecting environment	4.18	4.04	3.5
	Saving money	3.97	3.9	1.75
	Having continuous energy access	3.63	3.67	−1
	Improving the comfort temperature	2.59	2.69	−2.5
	Making my country energy-independent	4.01	4.13	−3
	Contributing to my country to have a more sustainable and efficient energy system.	4.3	4.38	−2
**Subjective norms**				
	Influence of family and friends	2.83	2.61	5.5
	Influence of my neighbours	2.69	2.46	5.75
	Influence of my colleagues	3.08	2.81	6.75
	Influence of me on my friends and family	2.77	2.69	2
**Perceived behavioural control**				
	Access to knowledge	3.67	3.54	3.25
	Affordable (money)	2.93	3.04	−2.75
	Enough time	2.67	2.73	−1.5
	Control of myself	2.51	2.3	5.25
**Personal moral norms**				
	I have a moral responsibility	4	3.61	9.75
**Descriptive norms**				
	My family members/friends did	3.55	3.13	10.5
	My neighbours and fellow citizens did	3.56	3.13	10.75
	My colleagues did	3.7	3.2	12.5
**Intention and behaviour**				
	Willingness to	4.28	3.99	7.25
	I will make an effort to	4.03	3.81	5.5

a $Difference\;in\;percentages=(\frac{\;{Average\;likert\;score}_{NL}-\;{Average\;likert\;score}_{EU-NL}}{{Average\;likert\;score}_{NL}}$) × 100.

As Table 5 demonstrates, the Dutch respondents have a relatively higher willingness to reduce their natural gas consumption compared to the rest of the sample. As the differences are presented in Table 5, subjective norms and personal descriptive norms have the most significant differences, potentially the main reason for the Dutch respondents' higher willingness and effort. These results are in line with the findings of studies such as [25], which demonstrate the environmentally friendly behaviour of Dutch households. In addition, the public Dutch perception towards gas quakes [60], high energy prices [61], their environmental concern [55] and collaborative culture [29] might be the reasons for the demonstrated differences. Although the number of respondents is not significantly high, these insights highlight the importance of such components in reducing natural gas consumption behaviours.

### 3.5 Sources of natural gas consumption

In the final part, the questioner posed questions about the sources of natural gas imports. As presented in Table 6, by scoring an average of 3.56, respondents showed that they highly care and want to know where the natural gas comes from. Furthermore, the majority of the respondents prefer the lowest possible natural gas imports. Such results confirm the previous results (see Sections 4.2. and 4.3.) about the importance of the country's energy-independence concerns.

**Table 6:** Natural gas imports.

Statements	The average score of respondents on the 5-Likert-type scale
**It is important for me to import as little natural gas as possible from another country.**	3.77
**It is important for me to consume natural gas extracted from my country of residence.**	2.78
**It is important for me to know the country's source of the natural gas that I consume for heating my house.**	3.56

**Fig. 3:**
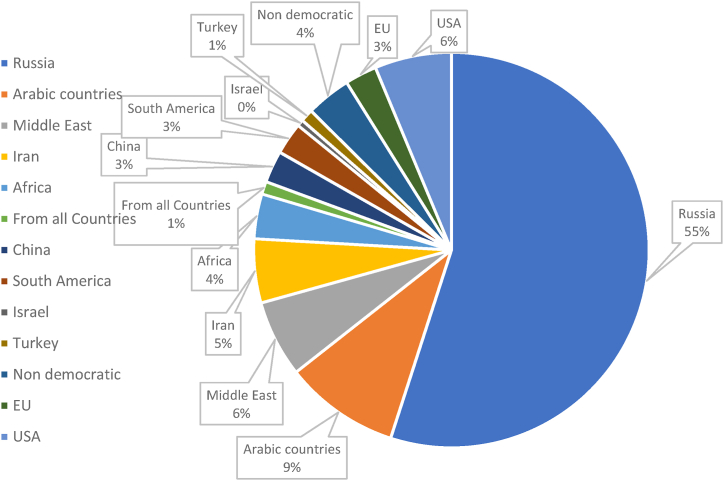
Regions and countries for avoiding natural gas imports

The following non-mandatory question was also posed: "Please mention a country/region from which you do not want the natural gas to be imported", which is posed to map out the opposing opinion towards a specific country or region to import the natural gas from European perception. Fig. 3 presents the percentage of the distribution of respondents for this question.

As Fig. 3 demonstrates, a significant percentage of respondents (55 %, 137 respondents) do not want their natural gas imported from Russia. This can be a response to the ongoing crisis in Eastern Europe and its related consequences and concerns, such as the energy crisis in the EU and worldwide, energy supply concerns, and humanitarian concerns. After Russia, with a considerable gap, the following countries and regions are the least favourable ones to import natural gas from: (i) Arab countries (e.g. Saudi Arabia, Qatar and Iraq), (ii) Middle Eastern countries, (iii) USA, and (iv) Iran. The respondents' Humanitarian concerns could potentially contribute to these results, as also in this line, without specifying a specific region/country, 4 % of respondents indicated that they do not want their natural gas imported from non-democratic countries. Furthermore, 3 % of respondents indicated that they do not want any imports outside EU borders, which potentially demonstrates the importance of energy independence for these respondents. On the other hand, 1 % of respondents indicated that they do not want natural gas at all, which is mainly due to environmental and independence concerns. Reasons such as "I want renewable heating" and "Any import is ultimately a dependency" are indicated.

## 4 Discussions and policy directions

The study collected and analysed data on intentions and behaviours for reducing natural gas consumption and import in Europe (EU) by conducting a questionnaire among highly educated individuals. The questionnaire consists of statements that the respondent must rate based on the level of agreement/disagreement with each statement. These statements represent different variables (e.g., environmental concerns, economic concerns and access to knowledge) connected to five components of the extended version of the theory of planned behaviour (TPB) and are on a 5-Likert type scale.

The results delineated the respondents' positive perception of highly educated individuals reducing natural gas consumption. The results showed that highly educated individuals perceive the current energy system as not leading to the comfort or satisfaction of the households, while it is perceived as highly available. Furthermore, 21 % of the respondents identified as households facing energy poverty. Considering that the sample is based on highly educated individuals (usually with higher and stable incomes), such results can be translated as the urgent need for transformation in the current energy system to avoid European energy poverty.

On the other hand, the study delineates the importance of moral responsibility and environmental and national concerns in reducing natural gas consumption decision-making. Although this study's focus is on natural gas consumption reduction and imports, its findings on the importance of such factors are in line with studies such as [[40], [41], [42]], which are focused on the renewable energy system adoption and investing in energy transition. Considering the correlations, environmental concerns and moral responsibility (a variable of personal descriptive norms) show the strongest correlations with the willingness and make an effort to reduce natural gas consumption. In contrast, the respondents do not feel they have any control over reducing their natural gas consumption and the thermal energy transition as a whole. These results can be translated as such variables (particularly having control) are the main barriers to reducing natural gas consumption. Furthermore, the study showed personal descriptive norms have more influence than subjective norms. Therefore, through concrete and transparent discussions about their energy-related behaviours, individual households could potentially encourage each other to reduce natural gas consumption and contribute to the (thermal) energy transition.

Furthermore, respondents showed serious concerns towards the source of natural gas, with a strong preference against Russia as the importing source. The respondents also clearly preferred not to import natural gas from non-democratic and non-humanitarian countries. Such results highlight that availability and affordability are not the only concerns and attributes that motivate people to change their behaviour towards a more sustainable behaviour (i.e., reducing natural gas consumption), but actually, social concerns (e.g. humanitarian concerns) along with environmental concerns also play a crucial role in such a context. The timeframe of the questionnaire is at the beginning of 2023, and the strong opposition to importing natural gas from Russia could be seen as a response of the highly educated individuals (i.e., respondents) to the ongoing crisis in Eastern Europe.

These insights contribute to studies such as [41,42], which used TPB to investigate the factors influencing individuals' attitudes and behaviour towards renewable energy systems. The study and its insights contribute to the literature on energy behaviour and individuals' decision-making processes. On the other hand, the study strengthens studies such as [9,25,62], which explore the alternatives for reducing natural gas consumption and imports in Europe. Furthermore, the results add to the literature on European strategies for reducing natural gas imports from Russia (e.g., Refs. [36,37,63]) as a response to the ongoing crisis in Eastern Europe and overall European natural gas consumption (e.g., Refs. [[64], [65], [66]]).

### 4.1 Recommendations

Considering the socio-demographic characteristics of the sample (as explained in Section 4), the thermal energy infrastructure (and natural gas purposed for heating individual households) requires going under significant transformation. The results from previous sections can be translated into detailed recommendations for policy-makers and individual households as follows.

❖ Policy-makers are urged to empower, inform, and involve individual households in the decision-making processes related to natural gas imports and consumption, as this is the perceived control behaviour variable that blocks the reduction of natural gas consumption.
❖ Economic indicators are overrated. As the study showed, environmental concerns and moral responsibilities have higher impacts. Therefore, all actors, particularly policy-makers and individual households, must promote and include such variables in their decision-making processes.
❖ The source of natural gas imports is important for the individual households. In particular, a significant number of respondents opposed importing natural gas from Russia. The respondents' perception is also highly against alternatives such as the United States, the Middle Eastern and Arab countries for importing natural gas.
❖ Environmental, humanitarian and energy-independence concerns need to be included in higher policy-making for energy strategies (e.g. energy imports and infrastructure planning). Such variables influence individual households and (change) their energy-related behaviours.
❖ Lastly, all the involved actors, particularly policy-makers, are urged to take action towards facilitating and contributing to the thermal energy transition, as the individuals indicated a high willingness to and make an effort to reduce their natural gas consumption.

### 4.2 Limitations

Although the study demonstrated a new application of the (extended version of) TPB theory and brought new insights to light for the stakeholders, particularly for energy policy-makers, it has certain limitations that need to be highlighted and considered.

The first limitation is the research method, namely the online questionnaire. As explained in Section 3, this method was used to collect and analyse empirical data on intentions and behaviours for natural gas consumption reductions. However, the study does not include the required actions, scenario planning, and technical and institutional conditions for such intentions and behaviours. Therefore, combining the findings with other research methods, such as interviews, serious gaming, district choice modelling, and equilibrium modelling, could be beneficial. As studies such as [42,43] demonstrated, computational social simulation approaches, such as agent-based modelling, could also be useful for studying energy-related behaviours and consumption.

The second limitation is also related to the online data collection approach. Although such an approach was chosen to reach a broader audience, it has certain technological requirements for participants (e.g., access to the internet through smartphones or computers). Such limitation could potentially bias the results, as certain social groups cannot access such technologies. For future research, it would be insightful to study the same topic through other approaches (e.g., paper-based questionnaires or interviews) to expand and diversify the sample, compare the results, and have more concrete insights.

The research also deliberately focused on highly educated individuals as pioneers and innovative individuals willing to change their behaviour. Although this sample brought meaningful insights to light for tackling the energy-related behaviour for facilitating the energy transition, for future research, it is meaningful to consider the public perception and include diverse households with various characteristics to have a more detailed and realistic overview of public perception towards reducing natural gas consumption and import sources.

The number of participants and the geographical focus of the study is the fourth limitation. The study is focused on the European Union (EU), emphasising the Netherlands. Although the EU (and the Netherlands) provide(s) an opportunity to explore the perception towards natural gas reduction (as elaborated in Sections 1, 2 and 3), these choices are a limitation, as they are from a specific context. Exploring similar questions in the context of other countries and regions (e.g. Africa or Asia) could lead to different results. Thus, it is insightful for future research to explore the questions in other contexts. Furthermore, the number of respondents is limited to 257 and could be increased to increase the sample size, potentially leading to confirming current outcomes. Also, largening the sample size could be beneficial in analysing the correlation of respondents' socio-demographic characteristics with their behaviours.

The fifth limitation is the theoretical background. By using the theory of planned behaviour (TPB), the study provided insights into the perception and behaviour of individuals about reducing natural gas consumption. However, applying theories such as Ostrom's Collective Action theory [67], the multi-level perspective [68], and the behavioural reasoning theory [69] could lead to more detailed insights regarding the decision-making processes and the diffusion of alternative energy systems (as a substitute) for reducing natural gas consumption.

Sixthly, the data is collected and analysed for limited variables (in total, 20 variables), structured according to the extended version of TPB. Although these variables are based on previous studies and the related results are discussed in detail (see Sections 1 and 2), other variables could also be included (e.g. variables related to energy justice). Including such variables through the TPB could contribute to painting the bigger picture of behaviours related to reducing natural gas consumption.

Lastly, the study is focused on the perception towards natural gas consumption without diving into details of alternative energy systems, which was out of the scope of this research. The study provided detailed insights on intentions and behaviours related to natural gas consumption reduction without connecting the findings with solutions such as (local) renewable thermal energy systems (e.g., local renewable energy systems based on solar energy [70]) and increasing insulation, which could be beneficial. However, as elaborated in studies such as [71], demand reduction strategies and electrification can significantly affect the (thermal) energy transition. Furthermore, considering and translating the insights into the context of thermal energy communities as collective, renewable and local energy systems [62,72] could bring further insights to light.

## 5 Conclusions

Climate change and the need for energy transition highlighted the need to reduce the consumption of different fossil fuels. In addition to the climate change concerns, the energy crisis resulting from the ongoing crisis in Eastern Europe significantly highlighted the need to reduce natural gas consumption in Europe (EU). Such reduction requires the behavioural change of individual Europeans as final energy consumers, particularly as individual households are responsible for a considerable share of total energy consumption within the EU. Therefore, this study aimed to explore the perception towards reducing natural gas consumption and imports. The research collected empirical data from 257 highly educated EU respondents by conducting a questionnaire. To study and explore the intentions and behaviours towards natural gas consumption constructively, the questionnaire and the analysis are based on an extended version of the theory of planned behaviour (TPB), where the intentions and, eventually, the behaviours are influenced based on five main components, namely: (i) Attitudes, (ii) Subjective norms, (iii) Perceived behaviour control, (iv) Personal moral norms, (v) Descriptive norms.

The results demonstrated a relatively high willingness and making an effort to reduce natural gas consumption among the respondents. Respondents indicated that although the current heating energy systems (which are mainly based on natural gas consumption in the EU) are mostly available, they are not leading to a comfortable and satisfying living environment. 21 % of respondents face energy poverty, a significant number based on EU standards. All these highlight the need for a drastic change in current heating systems.

The importance of the variables related to attitudes and personal moral norms was highlighted for willingness to reduce natural gas consumption. Particularly, moral responsibility and environmental concerns were found to be highly correlated with the desired intention for the behaviour (i.e., reducing natural gas consumption). In addition to these two variables, the descriptive norm's variables showed a higher correlation with reducing natural gas consumption rather than only being willing to reduce natural gas consumption. This can be translated as the importance of such variables (e.g., realising others have done it already) to make the willingness to have a more tangible output, namely making an effort to reduce natural gas consumption.

The study also showed that perceived behaviour control variables (such as available knowledge, affordability, having time and having control) are not the most correlated and highly-scored variables overall. However, within this context, respondents indicated that they know enough about reducing natural gas consumption while lacking control over such decisions. The low score and correlation of affordability and having time variables can be translated as their less influence on reducing natural gas consumption behaviour. Respondents also showed that they care about the sources of natural gas imports and do not want natural gas imports from specific countries (due to environmental, independence and humanitarian reasons). These all highlight that moral responsibilities and attitudes influence such behaviour rather than only economic and accessibility concerns.

The concrete results that the study brought to light contributed to energy behaviour and energy policy literature. These results could be translated as concrete recommendations for specific actors, as elaborated in 4.1. The study scientifically contributed to the literature by presenting a novel application of (the extended version of) the Theory of Planned Behaviour (TPB) in complex socio-technical (energy) systems, which also confirmed the applicability of the extended version as a useful tool for studying energy-related behaviour. Furthermore, besides contributing to energy-related literature, the study adds to the broader complex socio-technical systems by demonstrating the connection between individual decisions and higher-level policy-making.

Although the study provided insights into natural gas consumption reduction in the EU by employing a structured approach, it is important to keep in mind its limitations and the fact that it is the first attempt of its kind. Therefore, the study should be seen more as a starting point for its aims with a structured theoretical background. Along with the societal and scientific contributions in the energy field, the current work also contributes to the literature by bridging the behaviour of individuals to the higher level of policy-making. Therefore, considering the mentioned limitations, such contributions could be considered avenues for future research.

## Data availability statement

The related data will be made available on request, followed by the European General Data Protection Regulation (GDPR) guidelines.

## CRediT authorship contribution statement

**Javanshir Fouladvand:** Writing – original draft, Visualization, Validation, Supervision, Project administration, Methodology, Formal analysis, Data curation, Conceptualization. **Francesco Fiori:** Writing – review & editing, Visualization, Methodology, Formal analysis, Data curation. **Özge Okur:** Writing – review & editing, Validation, Project administration, Methodology, Conceptualization.

## Declaration of competing interest

The authors declare that they have no known competing financial interests or personal relationships that could have appeared to influence the work reported in this paper.

## Figures and Tables

**Table:** 

Statement	Level of Agreement
	1 to 5
The current heating system is always available.	
The current heating system is always affordable.	
Due to the current heating system, my accommodation is always comfortable.	
The current heating system is environmentally friendly.	
I am satisfied with the current heating system.	

**Table:** 

TPB components	Statements
**Attitude towards reducing natural gas**	I think reducing natural gas consumption for heating in my house is significant to protect the environment.
	I think reducing natural gas consumption for heating in my house is useful to save money.
	I think reducing natural gas consumption for heating in my house is valuable for continuous energy access.
	I think reducing natural gas consumption for heating in my house is important to improve the comfort temperature.
	I think reducing natural gas consumption for heating in my house is important for my country to become energy independent.
	I think reducing natural gas consumption will lead my country to achieve the energy transition's goals.
**Subjective norm**	Most of my family and friends think that I should reduce natural gas consumption in my house.
	Most of my neighbours think that I should reduce natural gas consumption in my house.
	Most of my colleagues think I should reduce my natural gas consumption in my house.
	If I reduce my natural gas consumption, then most people who are important to me also reduce their natural gas consumption.
**Perceived behaviour control**	I can find (already have) the needed knowledge and skills to reduce our natural gas consumption for heating my house.
	Reducing natural gas consumption for heating in my house is financially affordable.
	I have sufficient time to work on reducing natural gas consumption for heating in my house.
	Whether or not to reduce natural gas consumption for heating in my house is completely up to me.
**Personal moral norm**	I think I have a moral responsibility to reduce my natural gas consumption in my house.
	I would feel happy/proud if I reduced my natural gas consumption for heating my house.
**Descriptive norm**	A number of my family members/friends have reduced their natural gas consumption in their households.
	A number of my neighbours and fellow citizens have reduced natural gas consumption in their households.
	A number of my colleagues have reduced natural gas consumption in their households.
**Reducing natural gas intention**	I am willing to reduce natural gas consumption for heating in my house.
	I will make an effort to reduce natural gas consumption for heating in my house.

## Appendix A Survey

### Background information

❖ How do you indicate your gender?
  a. Male
  b. Female
  c. Non-binary/Third gender
  d. Prefer not to say
❖ How old are you?
  a. <18
  b. 18-24
  c. 25-34
  d. 35-49
  e. 50-64
  f. >65
❖ What is your country of residence?
❖ Is the country of your residence the same as your national country?
❖ What is your highest education degree?
  a. Elementary School
  b. High School
  c. Professional training (practical skills)
  d. Bachelor or college
  e. Master
  f. PhD
❖ What is your employment status?
  a. Part-time employed (including self-employed)
  b. Full-time employed (including self-employed)
  c. Unemployed
  d. Retired
  e. Student
  f. Other
❖ How many people reside in your residence?
  a. Only you
  b. You and your partner
  c. Family house (2–5 people)
  d. Family House (more than 5 people)
  e. Shared House (2–5 people)
  f. Shared House (more than 5 people)
❖ Which of the following best describes where you live?
  a. Rural area
  b. Urban area, out of the city centre
  c. Urban area, inside the city centre
❖ How would you describe your wage in comparison with your country's average? (You can take as examples **the average gross wage** of these countries)
Netherlands: €56,000; Italy: €34,500, Poland: zł80,500, Romania: RON705,900 Canada: C$69,000; Brazil: R$40,300, South Korea: ₩46,520,000, Japan: ¥ 5,450,900, Egypt: EGP95,300, South Africa: R120,000, India: ₹186,000, Iran: IRR117,022,000,
  a. Much higher than the average
  b. A bit higher than the average
  c. Similar to the average
  d. A bit lower than the average
  e. Much lower than the average

### Current energy system

❖ What percentage of your monthly **net income** spend on your energy bills (e.g. electricity bills and natural gas bills)?
  a. Less than 5 %
  b. 5 %–10 %
  c. 10 %–15 %
  d. 15 % to 20 %
  e. Higher than 20 %
  f. I do not know
❖ What is your current heating/cooling energy system in your residence? (Multiple answers possible)
  a) National grid, based on natural gas/oil (e.g. CV and natural gas boilers for your building)
  b) Renewable district heating for the whole neighbourhood (e.g. CHP, geothermal energy)
  c) Individual renewable energy systems (e.g. solar thermal, heat pump, wood pellet for your building)
  d) Others
  e) I do not know
❖ On a scale from 1 to 5, please indicate the level of your agreement with these statements concerning the current energy use for the "heating/cooling energy source and system" in your residence. (1: Strongly disagree, 5: Strongly agree)

On a scale from 1 to 5, please indicate the level of your agreement with these statements concerning your perception towards natural gas consumption reduction in your residence. (1: Strongly disagree, 5: Strongly agree).

### Natural gas independence

On a scale from 1 to 5, please indicate the level of your agreement with these statements concerning the natural gas imports being consumed in your residence. (1: Strongly disagree, 5: Strongly agree).

❖ It is important for me to import as little natural gas as possible from another country.
❖ It is important for me to consume natural gas extracted from my country of residence.
❖ It is important for me to know the country's source of the natural gas that I consume for heating my house.

If you prefer, please mention the country/region from which you do not want the natural gas to be imported.
